# Microfluidic and MEMS-Based Biosensing Platforms for Fungal Respiratory Infections in Immunocompromised Patients: Toward Rapid, Specific, and Minimally Invasive Diagnosis

**DOI:** 10.3390/bios16050281

**Published:** 2026-05-12

**Authors:** Vasiliki E. Georgakopoulou, Vassiliki C. Pitiriga

**Affiliations:** 1 Department of Pathophysiology, Laiko General Hospital, National and Kapodistrian University of Athens, 11527 Athens, Greece; 2 Department of Microbiology, Medical School of Athens, National and Kapodistrian University of Athens, 75 Mikras Asias Street, 11527 Athens, Greece

**Keywords:** fungal infections, respiratory infections, biosensors, invasive fungal respiratory infections, immunocompromised, microfluidic

## Abstract

Invasive fungal respiratory infections (IFRIs) remain a major cause of morbidity and mortality among immunocompromised patients, yet diagnosis continues to be hindered by nonspecific clinical features, limited sample accessibility, and the poor sensitivity or specificity of conventional tests. Microfluidic and microelectromechanical systems (MEMS)-based biosensing platforms have emerged as promising alternatives, enabling rapid, minimally invasive, and highly specific detection of fungal pathogens and host responses. Microfluidic nucleic acid and antigen assays allow on-chip amplification and immunodetection with reduced sample volumes and turnaround times, while CRISPR-enhanced systems further improve analytical sensitivity. Parallel advances in host response profiling—including transcriptomic, proteomic, and cytokine-based signatures—have demonstrated feasibility for integration into lab-on-a-chip platforms. MEMS-based technologies extend this potential by facilitating real-time analysis of exhaled volatile organic compounds, mechanical biosensing of fungal DNA and antigens, and in situ monitoring of device-associated biofilms. Translational studies highlight potential applications across intensive care, hematology–oncology, and transplant settings, as well as in outpatient monitoring of high-risk populations. However, several challenges remain, including limited multicenter validation, matrix-related biofouling effects, and a lack of standardization in fungal biomarker panels. Future directions include AI-driven interpretation of multianalyte data, multiplexed integration of host and pathogen markers, and development of fully cartridge-based systems for near-patient deployment. Collectively, these innovations may shift fungal diagnostics toward earlier, more precise, and patient-tailored interventions, improving outcomes in vulnerable populations.

## 1. Introduction

Invasive fungal respiratory infections (IFRIs) represent a significant clinical challenge, particularly among immunocompromised individuals, and are associated with high morbidity and mortality. These infections, predominantly caused by *Aspergillus* spp., *Candida* spp., *Cryptococcus neoformans*, and mucormycetes, are increasingly recognized in patients with compromised host defenses [1,2]. The lungs are a frequent portal of entry for these opportunistic pathogens, and pulmonary involvement can rapidly progress to disseminated disease if not promptly diagnosed and treated [3].

Certain populations are especially vulnerable to IFRIs, including recipients of hematopoietic stem cell transplantation (HSCT) and solid organ transplantation (SOT), patients with hematologic malignancies, individuals undergoing prolonged corticosteroid or immunosuppressive therapy, and those in intensive care units (ICUs) [4,5,6]. In recent years, the COVID-19 pandemic has introduced an additional at-risk group, as severe SARS-CoV-2 infection has been linked to increased susceptibility to secondary fungal infections such as COVID-19-associated pulmonary aspergillosis (CAPA) and mucormycosis [7,8,9]. The convergence of immunosuppression, pulmonary damage, and altered host-microbiome interactions in these settings further complicates timely diagnosis.

A major obstacle in the clinical management of IFRIs remains the delay in diagnosis. Conventional diagnostic methods—including culture, histopathology, serologic markers such as galactomannan and β-D-glucan, and imaging—are often time-consuming, lack sensitivity or specificity, and may require invasive sampling [10,11]. Given the nonspecific nature of early symptoms and radiographic findings, IFRIs frequently remain undiagnosed until advanced stages, when therapeutic interventions are less effective [12]. Diagnostic delays have been directly associated with increased mortality, underscoring the urgent need for novel tools capable of providing rapid and specific results at the point of care [13].

Conventional diagnostic methods for invasive fungal respiratory infections, although widely used, are limited not only by turnaround time but also by suboptimal analytical sensitivity and variable limits of detection. Fungal culture, while considered the reference standard, typically requires several days to yield results and demonstrates relatively low sensitivity, particularly in respiratory specimens where fungal burden may be minimal, with detection rates reported as low as 30–50% in proven cases [8,9]. Serological assays such as galactomannan enzyme-linked immunosorbent assay (ELISA) can detect circulating antigen at concentrations in the low ng/mL range, with reported sensitivities of approximately 60–90% depending on the patient population and sample type, whereas β-D-glucan (BDG) assays exhibit limits of detection in the pg/mL to low ng/mL range but lack specificity for fungal species [10,11,12]. Molecular methods, including conventional and quantitative polymerase chain reaction (PCR), offer improved analytical sensitivity, with detection thresholds as low as 10–100 genome copies per reaction under optimized conditions; however, their performance remains influenced by sample quality, presence of inhibitors, and lack of methodological standardization [14,15,16]. Collectively, these limitations highlight the need for diagnostic platforms that combine rapid turnaround with consistently low limits of detection and high clinical sensitivity across diverse specimen types.

Recent advances in biosensing technologies offer promising alternatives to traditional diagnostics. In particular, the integration of microfluidic systems and microelectromechanical systems (MEMS) into biosensing platforms has enabled the miniaturization and automation of complex laboratory procedures, making them more suitable for bedside or outpatient use [17,18]. These platforms allow for the manipulation of minute sample volumes, rapid analyte detection, and the potential for multiplexing, which are critical features in the diagnosis of fungal pathogens that require both speed and specificity [19].

This review aims to explore recent innovations in microfluidic and MEMS-based biosensing platforms tailored for the diagnosis of IFRIs in immunocompromised patients. It will examine current challenges in fungal diagnostics, the principles of microfluidic and MEMS technologies, and recent examples of biosensor development targeting fungal pathogens. Emphasis will be placed on systems that demonstrate potential for rapid, specific, and minimally invasive point-of-care applications, with a view toward facilitating early diagnosis and improving clinical outcomes in this vulnerable patient population.

## 2. Clinical Challenges in Diagnosing Fungal Respiratory Infections

The clinical diagnosis of IFRIs remains a formidable challenge, particularly in immunocompromised populations, where early detection is critical to prevent dissemination and death. IFRIs are most commonly caused by *Aspergillus fumigatus*, *Pneumocystis jirovecii*, and *Cryptococcus neoformans*, though the exact pathogen profile varies depending on the host’s immune status, underlying condition, and geographic context [20,21,22]. These pathogens can cause rapidly progressive pulmonary disease, yet often present with nonspecific symptoms that mimic other common respiratory conditions, including bacterial pneumonia and viral infections.

One of the key barriers to early diagnosis lies in the non-specificity of both clinical and radiological features. Fever, cough, dyspnea, and chest discomfort may occur, but are not pathognomonic. Radiological findings such as ground glass opacities, nodules with halo signs, or cavitary lesions are suggestive but not definitive for fungal infection, and may also be seen in malignancies, viral pneumonia, or drug-induced lung injury [23,24,25]. This lack of specificity complicates the diagnostic workup and often delays targeted antifungal therapy.

Another substantial difficulty relates to sample acquisition. Bronchoalveolar lavage (BAL) fluid remains a cornerstone for microbiological and biomarker testing, yet the procedure is frequently contraindicated in critically ill or severely hypoxemic patients due to its invasiveness and risk of decompensation [26]. Induced sputum may be unreliable in non-productive cough, and lung biopsies, although diagnostic, are rarely feasible outside of select cases due to their invasive nature and associated morbidity [7].

Current diagnostic methods suffer from significant limitations in sensitivity, specificity, or timeliness. Fungal cultures, although considered the gold standard, are notoriously slow and often yield negative results, particularly in patients who have received prior antifungal therapy or have low fungal burden [8]. The sensitivity of fungal cultures from BAL fluid or sputum in cases of proven invasive aspergillosis can be as low as 30–50% [9]. Furthermore, positive cultures may represent colonization rather than true infection, particularly in patients with underlying lung disease.

Non-culture-based methods, such as serological assays, have improved diagnostic capabilities but also pose interpretative challenges. Galactomannan (GM), a component of the *Aspergillus* cell wall, is widely used in serum and BAL samples, yet false positives may arise from dietary factors, concurrent antibiotic use (e.g., piperacillin–tazobactam), or cross-reactivity with other fungal species [10,11]. β-D-glucan (BDG), a panfungal biomarker, has broader sensitivity but limited specificity, making it less useful for differentiating between fungal species [12]. Additionally, both assays may yield false negatives in early infection stages or in patients receiving mold-active antifungals.

Imaging plays a crucial role in the diagnostic workup but is rarely definitive. Computed tomography (CT) findings may prompt suspicion for IFRI, but overlap with other causes of pulmonary infiltrates remains a persistent problem. As a result, many clinicians resort to empiric antifungal treatment in high-risk febrile neutropenic patients, which may lead to overtreatment, drug toxicity, increased costs, and antifungal resistance [13].

The cumulative effect of these limitations is a diagnostic landscape characterized by uncertainty, delayed intervention, and suboptimal outcomes. The need for diagnostic tools that are not only rapid and sensitive, but also minimally invasive and amenable to point-of-care deployment is thus both clear and urgent. In this context, biosensing platforms employing microfluidic and MEMS-based technologies offer a potentially transformative approach to IFRI detection, particularly if designed to integrate sample handling, pathogen detection, and result reporting in a compact format suitable for use in ICUs, transplant wards, or outpatient clinics.

## 3. Microfluidic Platforms for Fungal Pathogen Detection

### 3.1. Nucleic Acid Detection

Nucleic acid-based diagnostics have become the cornerstone of infectious disease detection due to their high sensitivity, specificity, and ability to identify pathogens at the species level. For fungal respiratory infections, molecular detection methods—particularly polymerase chain reaction (PCR) and isothermal amplification—offer significant advantages over culture and serological testing, especially when integrated into microfluidic platforms capable of rapid and automated analysis.

Microfluidic systems have been successfully adapted for on-chip PCR to detect fungal DNA in clinical specimens, notably *Aspergillus fumigatus*, *Pneumocystis jirovecii*, and *Cryptococcus neoformans*—the primary pathogens involved in IFRIs in immunocompromised patients [14,15,16]. Such devices can perform thermal cycling within a microchamber, using minimal sample volumes, and drastically reduce time-to-result compared to benchtop PCR systems. Moreover, microfluidic chips facilitate the parallelization of reactions, enabling multiplex detection of multiple fungal targets within a single cartridge [27].

Isothermal amplification techniques, such as loop-mediated isothermal amplification (LAMP), are particularly suited to point-of-care integration because they obviate the need for thermal cycling. LAMP assays targeting *Aspergillus* spp., *P. jirovecii*, and *C. neoformans* have demonstrated robust performance in clinical studies, with some achieving sensitivities and specificities comparable to conventional quantitative PCR (qPCR) [28,29,30]. Their incorporation into lab-on-a-chip platforms has enabled simplified workflows, making these tools promising candidates for use in resource-limited settings or bedside diagnostics [31].

Recent advances have also brought CRISPR-Cas systems into the microfluidic domain. CRISPR-based diagnostics, which rely on guide RNA-directed cleavage of target DNA or RNA sequences by Cas enzymes, have shown exceptional potential for fungal pathogen detection. When coupled with pre-amplification strategies such as LAMP or recombinase polymerase amplification (RPA), CRISPR-Cas12 and Cas13 platforms can achieve femtomolar sensitivity with high specificity for fungal nucleic acids [32].

Emerging studies have reported CRISPR–Cas-enabled microfluidic assays capable of distinguishing *Aspergillus fumigatus* from other *Aspergillus* species within approximately 1 h using respiratory samples such as bronchoalveolar lavage fluid, with limits of detection down to a few genome copies per reaction and diagnostic sensitivity and specificity in the high 80–100% range when compared with conventional PCR or sequencing-based reference methods [33,34].

In most of these microfluidic nucleic acid assays, analytical performance is underpinned by the use of multicopy or species-specific genomic targets that maximize signal at low fungal burdens. PCR- and LAMP-based platforms for *Aspergillus* spp. preferentially amplify ribosomal DNA loci (18S rRNA, 28S rRNA and internal transcribed spacer regions) or species-specific genes of *A. fumigatus*, achieving limits of detection in the order of 10–100 genome copies per reaction, which corresponds to approximately 1–10 CFU/mL in spiked bronchoalveolar lavage (BAL) or sputum matrices in benchtop evaluations [24,25,26,27,28,29,30,31]. Microfluidic LAMP assays for *Pneumocystis jirovecii* typically target the mitochondrial large subunit (mtLSU) rRNA or other high-copy-number loci and report comparable detection thresholds (10–50 copies per reaction), allowing reliable detection at low fungal loads relevant to early pneumonia [28,29,30,31]. For *Cryptococcus neoformans*, lab-on-a-chip nucleic acid tests have focused on cryptococcal ribosomal regions or capsular-associated genes, with analytical sensitivities down to a few tens of copies per reaction in cerebrospinal fluid and serum [26,30,31].

When CRISPR–Cas12/13 readouts are coupled to upstream LAMP or RPA in these microfluidic platforms, reported detection limits for *A. fumigatus* DNA fall in the low copy-number range (approximately 3–10^2^ copies per reaction), with clinical evaluations in bronchoalveolar lavage fluid demonstrating sensitivities of about 96–100% and specificities of 90–100% when benchmarked against qPCR or culture-based standards [33,34].

A critical component of integrating nucleic acid detection into microfluidic biosensors is the inclusion of upstream sample processing steps. On-chip lysis, nucleic acid extraction, and purification remain technical bottlenecks but are increasingly being addressed through the incorporation of microvalves, electrokinetic fluid control, and surface-anchored magnetic beads [35,36]. Fully integrated devices combining sample input with lysis, purification, amplification, and detection have been developed for respiratory specimens such as BAL fluid or sputum, streamlining the entire workflow into a single cartridge-based system [37]. These closed-system microfluidic platforms not only minimize contamination risks but also eliminate the need for complex laboratory infrastructure.

### 3.2. On-Chip Sample Processing and Sample-to-Answer Integration

A central objective of point-of-care biosensing platforms is the realization of true “sample-to-answer” capability, whereby raw clinical specimens are processed and analyzed within a fully integrated system without the need for external laboratory steps. Despite this, sample pretreatment remains comparatively underdeveloped in many microfluidic and MEMS-based fungal diagnostic platforms, representing a critical translational bottleneck. This limitation is particularly relevant in the context of fungal respiratory infections, where both the biological properties of the pathogen and the physicochemical characteristics of the sample impose significant technical challenges. Fungal cells, including Aspergillus spp. and Candida spp., possess rigid and multilayered cell walls composed of chitin, β-glucans, and mannoproteins, which confer structural resistance to conventional lysis approaches and necessitate optimized mechanical, chemical, or enzymatic disruption strategies prior to nucleic acid or antigen detection. In parallel, respiratory specimens such as sputum and bronchoalveolar lavage fluid are highly viscous and heterogeneous, often requiring liquefaction, homogenization, and removal of cellular debris to ensure efficient analyte recovery and reproducible fluid handling within microchannels.

Recent advances in microfluidic engineering have begun to address these challenges through the integration of automated on-chip sample processing modules, including chemical lysis chambers, bead-based mechanical disruption, enzymatic digestion, and electrokinetic or magnetically assisted nucleic acid extraction systems [35,36,37]. Such approaches enable sequential processing steps—from raw sample input to purified analyte—within a closed cartridge, reducing contamination risk and operator dependency while improving turnaround time. Microfluidic platforms incorporating magnetic bead-based extraction and microvalve-controlled workflows have demonstrated the feasibility of fully integrated sample-to-answer systems for respiratory specimens, including BAL and sputum, although most remain at the prototype or small-cohort validation stage [35,36,37]. However, challenges persist, including incomplete lysis of fungal elements, variability in sample viscosity affecting flow dynamics, and potential loss of analyte during multi-step processing. Moreover, the lack of standardized protocols for on-chip liquefaction and preprocessing of respiratory samples limits cross-platform comparability and reproducibility. Consequently, further development and clinical validation of robust, automated sample preparation modules will be essential to unlock the full potential of point-of-care fungal biosensing and to ensure reliable performance in real-world clinical environments.

A particularly underemphasized yet critical bottleneck in on-chip sample processing for fungal diagnostics is the efficient disruption of the fungal cell wall, which is structurally complex and substantially more resilient than that of bacteria. Fungal cell walls are composed of highly organized layers of chitin, β-(1,3)- and β-(1,6)-glucans, and mannoproteins, conferring significant mechanical rigidity and chemical resistance [38,39]. As a result, lysis strategies commonly employed for bacterial cells—such as mild chemical detergents or thermal disruption—are often insufficient for fungal pathogens, leading to suboptimal nucleic acid or antigen recovery and reduced diagnostic sensitivity.

Within microfluidic platforms, three principal lysis approaches have been explored, each with distinct advantages and limitations in the context of lab-on-a-chip integration. Mechanical lysis methods, including bead beating, acoustic cavitation, and microfluidic constriction-based shear stress, are among the most effective for disrupting rigid fungal cell walls and can achieve high recovery yields [40]. However, these approaches often require complex chip architectures, increased energy input, and may introduce debris that interferes with downstream detection or fouls sensitive MEMS transducers. Enzymatic lysis, typically employing chitinases, glucanases, or lyticase, offers a more selective and biologically targeted strategy, preserving nucleic acid integrity and minimizing mechanical debris [41]. Nevertheless, enzymatic approaches are inherently slower, sensitive to reaction conditions (e.g., temperature, pH), and may be difficult to standardize in rapid point-of-care workflows. Chemical lysis methods, including the use of chaotropic agents, detergents, or alkaline buffers, provide rapid and easily integrable solutions within microfluidic cartridges, but their efficiency against fungal cell walls is variable and often incomplete unless combined with other modalities.

Consequently, hybrid lysis strategies that combine mechanical disruption with enzymatic or chemical enhancement are increasingly recognized as the most effective approach for fungal pathogens. For example, bead-assisted mechanical lysis followed by enzymatic digestion can significantly improve yield while reducing processing time. However, such combinations must be carefully optimized to ensure compatibility with downstream MEMS-based sensing elements. In particular, harsh chemical reagents or excessive particulate debris may compromise sensor surface integrity, alter resonance properties in mechanical biosensors, or introduce noise in electrochemical and impedance-based systems [42]. Therefore, an optimal lysis strategy in integrated microfluidic–MEMS platforms must balance efficient fungal cell disruption with preservation of analyte integrity and minimization of interference with transducer performance.

### 3.3. Fungal Antigen Detection

Antigen detection represents one of the most widely used approaches in the diagnosis of IFRIs, offering the ability to detect fungal cell wall components circulating in blood or respiratory fluids. Among the most clinically relevant fungal antigens are GM, BDG, and cryptococcal antigen (CrAg), each of which is a validated biomarker for *Aspergillus* spp., multiple pathogenic fungi, and *Cryptococcus neoformans*, respectively [43,44,45]. However, traditional antigen detection assays, including enzyme-linked immunosorbent assays (ELISAs) and lateral flow assays (LFAs), often require laboratory infrastructure, multistep procedures, and relatively long turnaround times. Recent advances in microfluidics and paper-based biosensing technologies are enabling the miniaturization and acceleration of antigen detection assays, facilitating rapid bedside testing and decentralized diagnostics.

Microfluidic ELISA platforms have been developed to overcome the limitations of conventional bench-top immunoassays by integrating fluid handling, antigen–antibody interactions, and signal readout within a compact chip. For galactomannan detection, microfluidic immunoassays have demonstrated comparable sensitivity to standard ELISA (e.g., Platelia™ Aspergillus), with significantly reduced assay time—often under 30 min [46,47,48]. However, these microfluidic galactomannan platforms have so far been evaluated primarily in small single-center studies or benchtop comparisons with conventional ELISA, and no fully cartridge-based, clinically validated point-of-care test has yet reached routine implementation.

Recent years have seen rapid progress in the miniaturization of fungal antigen assays through microfluidic and paper-based technologies. For galactomannan, microfluidic fluorescence immunosensors have demonstrated on-chip detection using ZnO nanoflower interfaces, highlighting the feasibility of sensitive, pump-free assays in lab-on-a-chip formats [49]. Broader advances in microfluidic point-of-care testing emphasize portable cartridge designs and automated flow control, enabling immunoassays to be performed outside centralized laboratories [50,51].

In the ZnO nanoflower (ZnONF)-based platform reported by Piguillem et al., the nanostructured ZnO coating plays a dual role, acting both as a high–surface-area scaffold for antibody immobilization and as an optical amplifier for the fluorescent signal [49]. ZnONFs are grown on a glass substrate inside the microchannel, creating a three-dimensional array of petals that markedly increases the density of immobilized anti-galactomannan antibodies and promotes efficient capture of Aspergillus galactomannan. After sample introduction, a classical sandwich immunoassay is performed: captured antigen is recognized by a fluorescently labeled secondary antibody, and the resulting immunocomplex accumulates on the ZnONF surface. The nanoflower morphology enhances excitation and emission by concentrating the local electric field and increasing scattering, thereby boosting fluorescence intensity compared to planar substrates. The device operates under capillary-driven flow without external pumps, achieves a total assay time of approximately 30 min, and reaches a limit of detection for galactomannan in the low ng/mL range, i.e., well below commonly used serum GM positivity thresholds [46,47,48,49].

**Figure 1:**
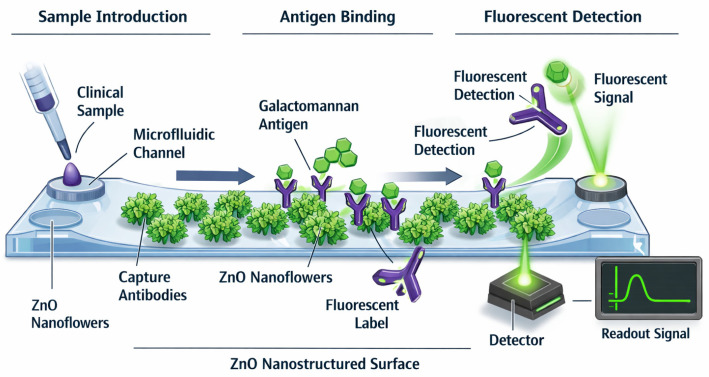
Microfluidic ZnO nanoflower (ZnONF)-based fluorescence immunosensor for galactomannan detection.

A schematic representation of this ZnONF-based microfluidic immunosensor is shown in Figure 1.

Schematic representation of a ZnONF-integrated microfluidic platform for the detection of Aspergillus galactomannan. ZnO nanoflowers grown within the microchannel provide a high–surface-area scaffold for immobilization of capture antibodies and enhance fluorescence signal through increased light scattering and local field amplification. Following sample introduction, galactomannan antigen binds to immobilized antibodies and is subsequently detected a fluorescently labeled secondary antibody in a sandwich immunoassay format. The nanostructured ZnONF surface improves sensitivity, enabling detection in the low ng/mL range within approximately 30 min. This platform demonstrates the potential of nanostructured microfluidic systems for rapid, sensitive, and point-of-care fungal diagnostics.

For BDG, traditionally detected with batch assays such as Fungitell™, research has focused on microfluidic paper-based devices capable of quantitative ELISA on paper substrates. To date, β-D-glucan detection on microfluidic and paper-based platforms remains at the preclinical or early pilot-testing stage, with most studies performed in spiked serum or small proof-of-concept cohorts rather than large, adjudicated clinical populations. Consequently, no BDG microfluidic device has yet progressed to regulatory approval or widespread clinical deployment, and these assays should currently be regarded as experimental complements to established batch tests. Such platforms reduce assay time, require only microliter sample volumes, and integrate colorimetric readouts that are compatible with smartphone imaging [52].

CrAg detection has also been transformed by digital and microfluidic approaches. The classical LFA remains a global standard, but artificial intelligence–driven mobile phone platforms have been shown to automatically interpret semi-quantitative CrAg LFAs, providing both qualitative and concentration-dependent readouts in real time [53,54]. This integration of digital readers with established LFAs represents an important step toward standardized bedside testing, particularly in resource-limited settings.

### 3.4. Host Response Profiling

Rapid, minimally invasive diagnosis of IFRIs in immunocompromised patients is hampered by low pathogen burden, sampling constraints (e.g., unsafe bronchoscopy), and slow mycological tests. Profiling the host response—the coordinated transcriptomic, proteomic and cellular immune signatures induced by infection—offers a complementary diagnostic axis that (i) can be measured from accessible matrices (whole blood, plasma/serum, finger prick), (ii) is amenable to lab-on-a-chip miniaturization for point-of-care use, and (iii) may also provide prognostic and treatment response information.

#### 3.4.1. Blood Transcriptomics and Circulating microRNAs

Whole-blood mRNA signatures can distinguish invasive aspergillosis (IA) from controls even under immunosuppression, provided analytic pipelines control for confounders introduced by steroids and chemotherapy. Steinbrink et al. derived and validated a conserved Aspergillus host response signature that retained diagnostic accuracy across immunosuppressive states, highlighting feasibility for clinical translation to rapid reverse transcription qPCR (RT-qPCR) panels on chip [54,55]. MicroRNA (miRNA) profiling is similarly promising: specific circulating miRNA patterns supported invasive aspergillosis (IA) diagnosis and prognosis in hematology/oncology cohorts, and miR-132/miR-155 are modulated in human myeloid cells by A. fumigatus [56,57,58]. These nucleic-acid markers align naturally with digital microfluidic RT-qPCR implementations that deliver multiplexed gene-expression readouts from microliter volumes in under an hour [59].

#### 3.4.2. Soluble Protein Biomarkers (Cytokines and Pattern Recognition-Linked Proteins)

Cytokine surrogates of Th1/Th17 inflammation—most consistently interleukin 6 (IL-6) and IL-8—are elevated in serum and BAL of hematologic patients with IA and have shown added diagnostic value alongside fungal PCR and lateral-flow assays [60,61,62]. Pentraxin-3 (PTX3), a humoral pattern recognition molecule produced by myeloid/endothelial cells, is increased in plasma/BAL in invasive pulmonary aspergillosis (IPA), augments galactomannan performance, and has emerging prognostic utility (mortality risk stratification; treatment response kinetics) [63,64,65,66]. Such proteins are ideal targets for multiplex immunoassays on microfluidic chips (bead-based, droplet-based, or electrochemical) capable of parallel IL-6/IL-8/PTX3 detection from a finger prick [67,68,69,70,71].

#### 3.4.3. Antigen-Specific T-Cell Responses

Aspergillus-reactive T-cell assays [Enzyme-Linked ImmunoSpot (ELISpot)/ELISA for interferon (IFN)-γ and IL-10] detect skewed Th1/Th2 responses during IA and have been explored for early diagnosis and immune monitoring in hematology and post-transplant settings [72,73,74]. Microfluidic single-cell platforms further enable high-throughput immune phenotyping (e.g., cytokine secretion profiling from individual PBMCs), reducing assay time and sample volume [68].

#### 3.4.4. Pneumocystis Jirovecii Pneumonia (PCP): Multi-Omics Host Signatures

In PCP, dynamic host responses across the disease course have been characterized by multi-omic and immunophenotyping studies, revealing distinct cytokine milieus and lymphocyte perturbations driven by the type/degree of immunosuppression [75,76,77]. While serum (1,3)-β-D-glucan remains pathogen-directed, host profiles (e.g., IL-6/IL-8, composite inflammatory indices) can improve risk prediction and may be adapted to rapid chips for rule-in/rule-out strategies when bronchoscopy is not feasible [61,77].

#### 3.4.5. Microfluidic/MEMS Enablement and Sample-to-Answer Workflows

Microfluidic and MEMS technologies now support integrated host response testing. Digital microfluidic RT-qPCR cartridges for multiplex host-gene signatures using whole blood, with on-cartridge extraction and 30–60 min turnaround [58]. Bead-array or droplet microfluidic immunoassays quantifying IL-6/IL-8/PTX3 (and additional cytokines) in <30 min from ≤50 µL serum, with analytical sensitivities in the low pg/mL range [66]. Electrochemical/photonic/MEMS sensors [e.g., silicon nanowire Field Effect Transistors (FETs)] for ultrasensitive IL-6 and related markers, compatible with portable readers for bedside testing [69,70].

A tiered algorithm is therefore feasible: (i) finger prick host response panel (IL-6/IL-8/PTX3 ± parsimonious mRNA signature) to triage high-risk immunocompromised patients with new pulmonary infiltrates; (ii) reflex pathogen-directed testing [e.g., plasma cell-free DNA (cfDNA)/Metagenomic Next-Generation Sequencing (mNGS) or respiratory PCR] when host test is positive or indeterminate; and (iii) serial host-marker monitoring (e.g., PTX3) to assess therapeutic response and prognosis [63,64,65].

## 4. MEMS-Based Tools in Fungal Diagnosis

### 4.1. VOC Detection from Exhaled Breath

Non-invasive detection of IPA through analysis of exhaled volatile organic compounds (VOCs) represents a rapidly evolving frontier in fungal diagnostics. *Aspergillus fumigatus* and other filamentous fungi emit unique VOCs during metabolic processes, including compounds such as 2-pentylfuran, α-trans-bergamotene, β-trans-bergamotene, and trans-geranylacetone, which can be detected in the breath of infected patients [78].

MEMS-based gas sensors are ideally suited for capturing these VOC signatures due to their small footprint, low power requirements, and capacity for real-time, label-free detection. Recent advances have enabled MEMS sensors to detect fungal VOCs with high sensitivity and selectivity at concentrations relevant to human breath. Arabi et al. developed MEMS-based bifurcation gas sensors capable of detecting volatile biomarkers such as hydrogen sulfide and formaldehyde, achieving detection thresholds as low as 1 ppm [79]. While these gases are not specific to fungi, the study highlights the precision and miniaturization potential applicable to fungal metabolite detection.

Electronic nose (e-nose) systems integrating MEMS chemiresistive sensors, quartz crystal microbalances (QCM), and field asymmetric ion mobility spectrometry (FAIMS) have demonstrated strong performance in identifying *Aspergillus*-specific VOC profiles. For example, a study using a porphyrin-coated QCM gas-sensor array exposed to headspace VOCs from cultured *Aspergillus* isolates correctly classified approximately 88% of samples in the training set and around 70% in an independent test set when discriminating *A. fumigatus*, *A. terreus* and other *Aspergillus* spp. [80]. In addition, an exhaled-breath electronic nose study in patients with cystic fibrosis colonized by *A. fumigatus* reported a cross-validated diagnostic accuracy of 89%, with a sensitivity of 78% and specificity of 94% for detecting airway colonization based on breath VOC patterns [81].

Perhaps the most clinically relevant progress comes from breathomics research. Koo et al. identified a panel of fungal VOCs from patients with confirmed IPA, including α- and β-trans-bergamotene and trans-geranylacetone, and reported clear separation between IPA and non-IPA groups using breath samples [78]. These findings have since been translated into patented biosensor designs, such as those described in US Patent US10227629B2, which proposes selective detection of IPA using MEMS sensors tuned to fungal sesquiterpenes [82].

To enhance specificity and usability, modern MEMS VOC biosensors incorporate functionalized sensing layers, such as metal oxide semiconductors (e.g., SnO_2_, ZnO), graphene derivatives, and molecularly imprinted polymers that preferentially bind fungal metabolites [83]. Sensor responses are typically interpreted using machine learning algorithms or multivariate statistical models (e.g., PCA, LDA, random forest), enabling differentiation of fungal infections from bacterial or viral mimics [84].

Clinical implementation of these systems is increasingly feasible, given their compatibility with handheld devices, breath collectors, and portable data analysis modules. A fluorescence-based MEMS microfluidic sensor capable of detecting fungal antigens in under 40 min using exhaled breath condensate or serum [85].

Despite these promising results, there is only partial concordance between VOC marker panels reported across different studies. Headspace experiments on cultured *Aspergillus* spp. often emphasize sesquiterpenes such as α- and β-trans-bergamotene and trans-geranylacetone, whereas clinical breathomics investigations in patients with invasive pulmonary aspergillosis identify broader signatures in which the relative contribution of these compounds varies with fungal burden, strain, antifungal treatment, host factors and environmental background [81,83,84]. Moreover, differences in sampling protocols, sorbent materials, gas-sensor types and data-processing pipelines contribute substantially to inter-study variability. As a result, current MEMS/electronic-nose platforms for Aspergillus VOC detection should be considered adjunctive tools whose algorithms and cut-offs require standardization and prospective multicenter validation before they can be widely adopted for routine clinical decision-making.

An additional limitation that warrants further consideration is the impact of co-infections and environmental background signals on the specificity of VOC-based biosensing platforms. Immunocompromised patients—particularly those in intensive care units or with hematologic malignancies—frequently present with polymicrobial infections involving bacteria, viruses, and fungi, each contributing distinct and potentially overlapping metabolic volatile profiles. As a result, VOC signatures attributed to fungal pathogens such as Aspergillus fumigatus may be confounded by concurrent bacterial colonization or infection, leading to reduced specificity and potential misclassification. For example, bacterial pathogens commonly associated with respiratory infections, including Pseudomonas aeruginosa and Staphylococcus aureus, produce volatile metabolites that may overlap with or mask fungal-derived compounds, thereby complicating pattern recognition by electronic nose systems or MEMS-based gas sensors.

In addition to biological confounders, environmental and exogenous factors—including ambient air composition, hospital ventilation systems, dietary influences, and prior antimicrobial or antifungal therapy—can further alter VOC profiles and introduce variability across measurements. Differences in sampling techniques, sorbent materials, sensor arrays, and data-processing pipelines also contribute to inter-study heterogeneity and limit reproducibility of VOC signatures across clinical settings [81,84]. These factors are particularly relevant in real-world conditions, where signal-to-noise ratios may be substantially lower than in controlled experimental environments. Although machine learning algorithms have been employed to enhance discrimination of complex VOC patterns, their performance is highly dependent on training datasets that may not adequately capture the diversity of polymicrobial and environmentally influenced clinical scenarios.

Taken together, these limitations underscore the need for standardized sampling protocols, inclusion of polymicrobial infection cohorts in validation studies, and development of robust multivariate models capable of distinguishing fungal-specific metabolic signatures from background noise. Integration of VOC analysis with complementary diagnostic modalities, such as host response biomarkers or nucleic acid detection, may further improve diagnostic accuracy and mitigate the impact of these confounding factors in immunocompromised populations.

A critical limitation of VOC-based fungal diagnostics that warrants further consideration is the impact of metabolic noise arising from polymicrobial co-infections, which are highly prevalent in immunocompromised patients. In clinical settings such as intensive care units and hematology–oncology wards, fungal pathogens frequently coexist with bacterial and viral organisms, each contributing distinct and potentially overlapping volatile metabolic signatures. For example, common bacterial respiratory pathogens such as *Pseudomonas aeruginosa* and *Staphylococcus aureus* produce alcohols, aldehydes, ketones, and sulfur-containing compounds that may partially overlap with fungal-derived VOCs, including sesquiterpenes and other secondary metabolites associated with *Aspergillus fumigatus* [86,87]. This overlap can significantly reduce diagnostic specificity and lead to signal ambiguity, particularly when fungal burden is low or when antifungal therapy alters metabolic output.

To address these challenges, modern VOC-based biosensing platforms increasingly rely on multi-sensor arrays—often referred to as electronic noses (e-noses)—combined with advanced pattern recognition algorithms. Rather than detecting a single biomarker, these systems capture a high-dimensional “breathprint” composed of the collective response of multiple semi-selective sensors, including metal oxide semiconductors, quartz crystal microbalances, and conductive polymer sensors [88,89]. Each sensor exhibits partial specificity to different chemical classes, and the combined response pattern allows discrimination between complex VOC mixtures. Machine learning techniques, such as principal component analysis (PCA), linear discriminant analysis (LDA), support vector machines (SVM), and random forest classifiers, are then applied to extract latent features and classify samples based on multidimensional signal profiles [90,91]. Importantly, these approaches enable the differentiation of fungal infection signatures from bacterial or viral backgrounds, even in the presence of overlapping metabolites, by identifying subtle but reproducible differences in the overall VOC pattern rather than relying on individual compounds. Furthermore, training these models on datasets that include polymicrobial infections and diverse clinical conditions improves robustness and generalizability in real-world settings. As such, the integration of multi-sensor architectures with machine learning-based signal processing represents a critical strategy for mitigating metabolic noise and enhancing the diagnostic accuracy of VOC-based MEMS platforms in complex clinical environments.

### 4.2. Mechanical Biosensors

Mechanical biosensors, including microcantilevers and resonant mass sensors, are increasingly recognized as powerful tools for the label-free and real-time detection of fungal biomarkers such as DNA, cell wall antigens, and even intact spores. These micro- and nanoelectromechanical systems [MEMS and Nanoelectromechanical Systems (NEMS)] operate by transducing biomolecular interactions into measurable mechanical signals—typically changes in mass, resonance frequency, or surface stress—without requiring enzymatic, fluorescent, or chemiluminescent labels. This intrinsic label-free operation, together with high mass resolution in the femtogram–picogram range, makes mechanical biosensors attractive candidates for compact, high-throughput diagnostic platforms targeting fungal infections in immunocompromised patients. At present, however, these mechanical biosensing platforms are confined to benchtop, preclinical studies and have not yet been evaluated in prospective clinical cohorts of immunocompromised patients.

Microcantilever biosensors function analogously to atomic force microscopy probes. In the static mode, the upper surface of the cantilever is functionalized with specific capture molecules—such as antibodies, oligonucleotide probes, or carbohydrate-recognizing receptors (e.g., Dectin-1)—while the opposite surface is left unmodified. Binding of the target analyte (fungal antigen, DNA, or polysaccharide) creates a differential surface stress between the coated and uncoated sides, leading to nanometer-scale bending of the cantilever that can be read out optically via a laser beam and position-sensitive detector, or electrically via integrated piezoresistive elements. In the dynamic (resonant) mode, the cantilever is driven at its resonance frequency; adsorption of biomolecules increases its effective mass and leads to a measurable downshift in resonance. Because the resonance frequency is highly sensitive to added mass, this configuration enables the detection of very low analyte concentrations from small sample volumes.

Several proof-of-concept studies have demonstrated the relevance of these platforms to fungal diagnostics. Cantilevers coated with galactomannan- or cryptococcal capsular polysaccharide–specific antibodies have been used to detect fungal antigens in serum-like matrices by monitoring deflection or resonance shifts, achieving analytical sensitivities down to the low pg/mL range in optimized settings [92,93]. Micro- and nanomechanical cantilever sensors functionalized with thiol-modified oligonucleotides on gold surfaces have detected DNA hybridization events corresponding to fungal gene targets; Mishra and Hegner reported femtomolar sensitivity for in situ hybridization in a model system, illustrating the potential for label-free nucleic acid detection at clinically relevant concentrations [94]. In another study, Nugaeva et al. functionalized gold-coated (and uncoated) silicon cantilevers with proteins to capture airborne fungal spores such as Aspergillus niger and monitored spore binding and subsequent growth via resonance frequency shifts, highlighting the ability of mechanical sensors to track dynamic fungal biomass changes in real time [95].

Mechanical biosensing concepts have also been extended to β-D-glucan detection. Liu et al. fabricated a composite film (Nafion–thionine–AuNP–chitosan) with immobilized Dectin-1 on an electroactive surface; binding of β-glucans from serum inhibited electron transfer and produced measurable amperometric suppression, with acceptable speed, accuracy, and stability as a surrogate of fungal burden [96]. Although this platform uses electrochemical rather than purely mechanical readout, it illustrates how pattern recognition receptors such as Dectin-1 can be integrated into miniaturized transducers for panfungal biomarker detection.

**Figure 2:**
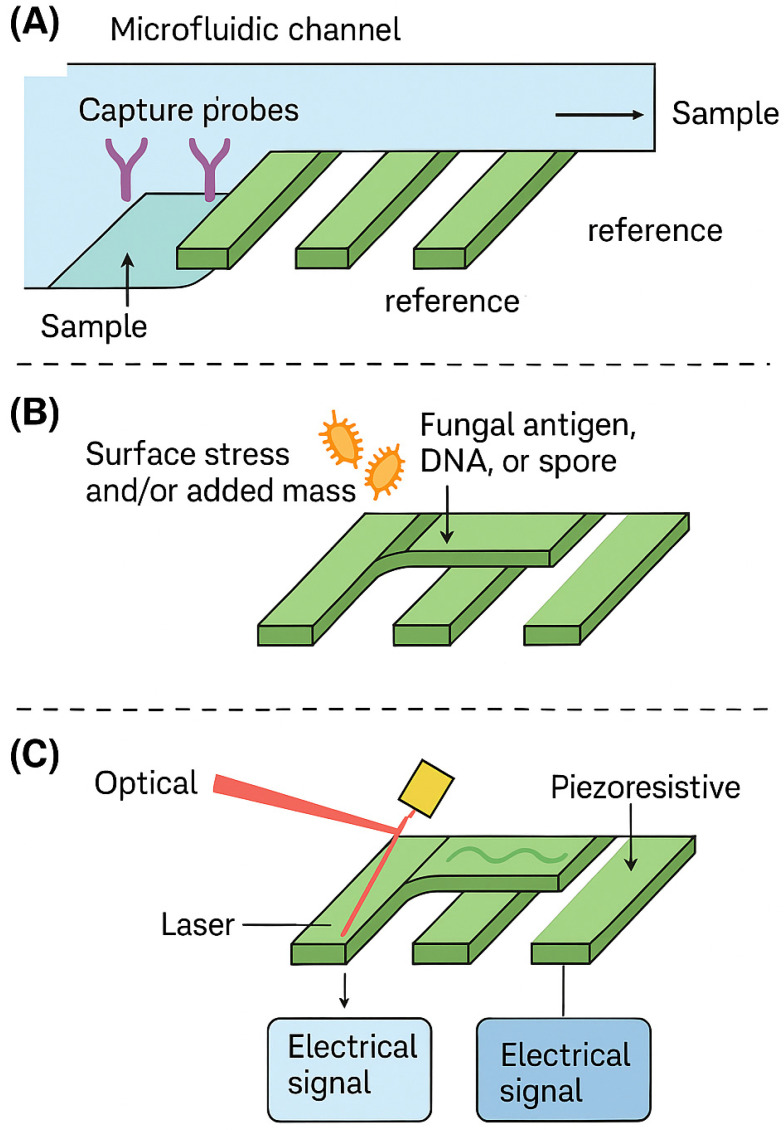
Schematic of a cantilever-based mechanical biosensor for fungal biomarkers. (**A**) Microcantilever array integrated into a microfluidic channel receiving serum or bronchoalveolar lavage (BAL) samples. Selected cantilevers are functionalized on their upper surface with capture probes (e.g., anti-galactomannan antibodies, anti-cryptococcal capsular antibodies, or fungal DNA oligonucleotide probes), while reference cantilevers remain unmodified. (**B**) The binding of fungal antigens, DNA, or spores induces surface stress and/or added mass, leading to static bending or a resonance frequency shift in the cantilever. (**C**) Optical (laser–photodiode) or piezoresistive readout converts the mechanical response into an electrical signal that can be processed by an external or handheld reader for quantitative, label-free detection of fungal infection.

In principle, cantilever arrays can be integrated with microfluidic sample handling to enable multiplex measurements (e.g., parallel detection of galactomannan, β-D-glucan, and species-specific DNA probes from a single bronchoalveolar lavage or serum sample) with turnaround times on the order of 10–30 min. A simplified schematic of a cantilever-based fungal biosensor, including surface functionalization, analyte binding, and optical/electrical readout, is presented in Figure 2.

Overall, microcantilever- and resonance-based fungal biosensors should therefore be viewed as early-stage research tools with excellent analytical performance but no clinical trial data to support routine diagnostic use at this time.

### 4.3. Device-Associated Fungal Biofilm Monitoring

Indwelling medical devices such as central venous catheters, urinary catheters, and endotracheal tubes are critical in the care of immunocompromised patients, yet they also serve as key substrates for fungal biofilm formation. *Candida albicans*, *Candida glabrata*, and *Aspergillus fumigatus* are among the most common fungal pathogens implicated in device-associated infections, which are notoriously resistant to antifungal therapy due to the protective biofilm matrix and altered metabolic state of the organisms [97].

Detection of biofilms on medical devices currently relies on indirect clinical signs (e.g., persistent fever despite therapy) or requires device removal for culture and microscopy, leading to diagnostic delay. In this context, MEMS-based biosensors embedded within or onto indwelling devices offer a transformative approach for real-time, in situ monitoring of fungal biofilm formation.

Detection of biofilms on medical devices currently relies on indirect clinical signs or device removal for culture and microscopy, leading to diagnostic delay. In this context, embedding MEMS-class biosensors on or within indwelling devices is a promising route for real-time, in situ monitoring of fungal colonization and biofilm growth. Early demonstrations with micromechanical cantilevers showed label-free detection of immobilized fungal cells via resonance frequency shifts—for example, Nugaeva et al. used antibody/lectin-functionalized silicon cantilever arrays to capture *Aspergillus niger* and *Candida* spp., with mass-loading readouts in dynamic mode [95,98]. For *Candida* spp. label-free impedance approaches can directly sense yeast attachment: a membrane-based electrochemical impedance sensor with anti-*Candida* capture enabled specific detection of *C. albicans* [99], and subsequent work has refined Electrochemical Impedance Spectroscopy (EIS) workflows for *Candida* spp. in clinical matrices (e.g., urine) with rapid, reagent-light measurements [100,101,102]. Recent reviews focused specifically on *Candida* spp. biosensing highlight optical and electrochemical platforms (including microfluidic/EIS integrations) as viable paths toward catheter-compatible monitoring of fungal biofilms, while noting that fully embedded, clinical-grade MEMS devices for fungi remain an active translational frontier [103,104].

## 5. Translational Applications in Immunocompromised Settings

The development of microfluidic and MEMS-based biosensing platforms holds substantial translational potential across a spectrum of clinical settings where immunocompromised patients are at risk of IFRIs. These technologies, capable of rapid, sensitive, and minimally invasive diagnostics, can be strategically deployed to address the unique challenges of fungal detection in ICUs, hematology-oncology departments, transplant centers, and even home-based environments.

In the ICU, ventilated patients often develop non-specific respiratory deterioration where rapid differentiation among bacterial, viral, and fungal etiologies is critical. Severe influenza and COVID-19 are established risk factors for invasive pulmonary aspergillosis [Influenza-Associated Pulmonary Aspergillosis (IAPA)/CAPA], with multicenter ICU data and consensus guidance underscoring excess morbidity and mortality when diagnosis is delayed [6,91]. Noninvasive fungal breath diagnostics show promise: targeted VOC profiles in exhaled breath distinguished proven/probable invasive aspergillosis with high sensitivity and specificity in a clinical cohort [79] and exhaled-breath condensate (EBC) GM testing has demonstrated feasibility against BAL GM in immunocompromised populations and in ventilated ICU patients [85,105].

In hematology–oncology patients during post-chemotherapy neutropenia, preemptive strategies anchored on serial serum GM plus high-resolution computed tomography (HRCT) have reduced empiric antifungal exposure while maintaining outcomes [106]. For earlier rule-in testing, BAL GM is analytically and clinically validated (systematic reviews/meta-analyses), and bedside Aspergillus GM LFAs—including reader-assisted formats—provide 15–45 min turnaround and have shown useful performance in cancer and ICU cohorts [107,108,109].

In transplant units, where febrile neutropenia is common and sample volumes are limited, pairing rapid serum/respiratory GM LFA with BAL GM (when feasible) supports risk-adapted antifungal initiation while minimizing unnecessary drug exposure [107,108,109]. Beyond the inpatient setting, longitudinal surveillance is emerging: electronic-nose breath profiling has detected *Aspergillus fumigatus* airway colonization in cystic fibrosis patients, suggesting a feasible outpatient monitoring pathway that could, after validation for invasive disease, enable earlier intervention post-discharge [81].

**Figure 3:**
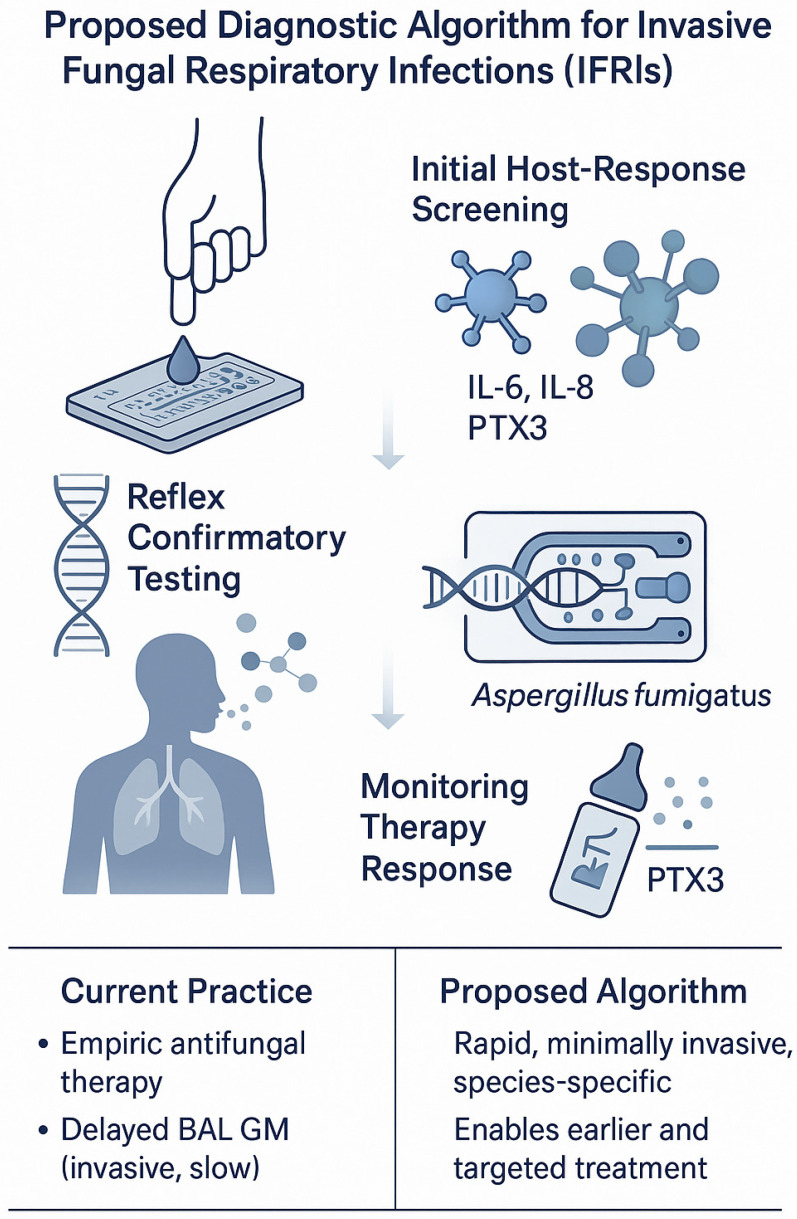
Proposed diagnostic algorithm for invasive fungal respiratory infections (IFRIs).

A proposed diagnostic algorithm integrating host response screening, reflex confirmatory testing, and monitoring is shown in Figure 3.

The algorithm outlines a tiered workflow for high-risk immunocompromised patients presenting with new pulmonary infiltrates. Step 1 (≈15–30 min; finger prick capillary whole blood): initial host response screening using a microfluidic immunoassay for IL-6, IL-8 and PTX3 [59,60,61,62,63,64,65]. The figure labels this step with its sample type and turnaround time, and indicates a clinical decision threshold of “host response panel above predefined high-risk cut-off” (based on published IL-6/IL-8/PTX3 thresholds in hematology/oncology cohorts). Step 2 (≈45–60 min; BAL or sputum/respiratory sample): reflex confirmatory testing using a CRISPR-enhanced microfluidic assay targeting *Aspergillus fumigatus* nucleic acids [32,33]. In the flow chart, this box is annotated with the respiratory sample type, expected detection time and the decision threshold “CRISPR *A. fumigatus* assay positive” (signal above assay-specific LOD in Table 1). Step 3 (≈15–45 min; serum and/or exhaled breath): therapy monitoring via serial PTX3 measurement and/or a MEMS-based volatile organic compound (VOC) breath sensor [63,64,65,78,79,80,81,82,83,84]. The figure indicates serum/exhaled-breath sampling, typical turnaround times, and the decision criterion “trend toward decreasing PTX3/normalization of VOC pattern” versus “persistent high-risk profile” to guide antifungal management. Compared with current practice—empiric antifungal therapy and delayed bronchoalveolar lavage galactomannan (BAL-GM) testing [10,11,12,13]—this tiered approach emphasizes earlier, minimally invasive and species-directed diagnosis. A comparative summary of the biosensor platforms including target analytes, sample types, performance metrics, and clinical validation stages, is provided in Table 1.

## 6. Clinical Validation Gaps and Real-World Performance

Although recent developments in microfluidic and MEMS-based fungal diagnostics have shown considerable technical promise, most platforms remain in early stages of clinical evaluation, with limited validation in immunocompromised patient populations. Many of the cited studies reporting high sensitivity and specificity for techniques such as microfluidic LAMP, CRISPR-based nucleic acid assays, and antigen detection methods—including galactomannan and β-D-glucan—are based on small single-center cohorts, often comprising fewer than 100 subjects, and frequently lack comparison against standardized clinical reference methods such as bronchoalveolar lavage galactomannan, PCR, or EORTC/MSG criteria [28,29,30,31,32,33,46,47,48,49].

These shortcomings largely reflect the developmental stage of most of the platforms discussed. Many microfluidic and MEMS-based assays are still in the engineering or proof-of-concept phase, where the primary objective has been to demonstrate analytical performance in well-controlled matrices (buffer, spiked serum or BAL) and to optimize fluidics, surface chemistry, and readout electronics. Access to sufficiently large, well-characterized clinical cohorts with adjudicated IFRI according to EORTC/MSG criteria is limited, and prospective collection of paired specimens for comparison with current gold standards (BAL galactomannan, culture, PCR, histopathology) is logistically complex and costly, particularly in immunocompromised populations. In addition, some platforms (e.g., breath VOC sensors, integrated catheter-based biofilm sensors) require dedicated sampling hardware or device modifications that are not yet approved for routine patient care, further restricting their evaluation in real-world settings. For these reasons, most assays have not yet undergone large-scale clinical trials or head-to-head comparison with established diagnostic methods, despite encouraging analytical data. As this work is a narrative review rather than a primary clinical trial, we summarize and critically appraise the available validation data but cannot compensate for the current lack of large multicenter studies.

While digital CrAg lateral flow assays have achieved broader clinical deployment, including semi-quantitative reader-assisted formats that demonstrate diagnostic accuracies exceeding 95% [53,54], other platforms such as microfluidic BDG assays, host response transcriptomic panels, multiplex cytokine biosensors, and MEMS-based volatile organic compound sensors remain in the preclinical or pilot-testing phase [52,54,55,56,57,58,59,60,61,62,63,64,65,66,78,79,80,81,82,83,84]. Most evaluations have been limited to controlled settings or proof-of-concept studies without robust real-world validation in hematology–oncology, transplant, or intensive care unit cohorts. In addition, the reported diagnostic performance is often confounded by heterogeneous sampling protocols, lack of antifungal exposure stratification, and absence of gold-standard comparators.

Mechanical and impedance-based biosensors targeting fungal DNA, cell wall antigens, or device-associated biofilms have demonstrated high analytical sensitivity in vitro but have not yet progressed to clinical trials [92,93,94,95,96,97,98,99,100,101,102,103]. No published studies currently support their performance in real clinical matrices such as serum, BAL fluid, or urine.

To support clinical translation, future research should focus on well-powered, prospective, multicenter studies using harmonized definitions of invasive fungal disease. Real-world performance assessments in diverse immunocompromised populations, alongside standardized cost-effectiveness and implementation analyses, will be essential to determine the true diagnostic value of these biosensing platforms.

A critical yet underemphasized dimension of clinical validation in emerging biosensing platforms for invasive fungal respiratory infections relates to their performance in complex biological matrices, rather than solely in buffered laboratory conditions or artificially spiked samples. While many microfluidic and MEMS-based systems demonstrate excellent analytical sensitivity and specificity under controlled conditions, these metrics may not translate into real-world diagnostic performance when assays are applied to clinical specimens such as sputum, BAL fluid, or serum. These matrices are inherently heterogeneous and contain high concentrations of mucins, host proteins, inflammatory cells, extracellular DNA, lipids, and commensal or pathogenic microorganisms, all of which can interfere with analyte capture, signal transduction, and fluid dynamics within microchannels. In particular, biofouling—the non-specific adsorption of biomolecules onto sensor surfaces—can significantly impair sensor stability, reduce effective binding capacity, and increase background noise, thereby compromising both sensitivity and specificity. This issue is especially pronounced in label-free platforms, including mechanical biosensors and electrochemical or impedance-based systems, where signal output is highly dependent on surface integrity and low-noise conditions [92,93,94,99,100,101,102]. Even nucleic acid-based and immunoassay-based microfluidic systems are not immune to matrix effects, as inhibitors present in respiratory samples may affect amplification efficiency, antigen–antibody interactions, or upstream sample processing steps such as lysis and extraction [35,36,37,46,47,48,49]. Notably, most currently available studies have evaluated these platforms using buffer solutions, spiked serum, or simplified BAL models, with limited validation in unprocessed or minimally processed clinical specimens, thereby raising concerns regarding their robustness and reproducibility in routine practice [28,29,30,31,32,33,46,47,48,49]. Furthermore, variability in sample viscosity, cellular content, and prior antifungal exposure may introduce additional sources of diagnostic heterogeneity that are rarely accounted for in early-stage evaluations. Although recent efforts to incorporate antifouling coatings, optimized microfluidic architectures, and upstream sample-conditioning modules have shown promise in mitigating these challenges, such approaches remain non-standardized and have not yet been systematically validated across platforms or patient populations [110,111,112]. Consequently, future clinical validation studies should move beyond conventional case–control designs and explicitly assess biosensor performance in authentic respiratory matrices, incorporating analyses of fouling kinetics, interference patterns, false-positive rates, and inter-operator variability. Demonstrating consistent diagnostic accuracy under these conditions will be essential to bridge the gap between analytical performance and true clinical utility in high-risk immunocompromised populations.

## 7. Regulatory and Economic Considerations

In addition to analytical and clinical performance, the successful translation of biosensor-based diagnostics for IFRIs depends on regulatory classification, reimbursement potential, and economic feasibility. In the United States, POC devices must meet the criteria for Clinical Laboratory Improvement Amendments (CLIA) waiver, which requires demonstration of analytical simplicity, minimal risk of erroneous results, and ease of use by non-laboratory personnel. To date, most fungal diagnostics, including the Platelia™ GM and Fungitell™ BDG assays, are not CLIA-waived and remain confined to centralized laboratories due to their technical complexity and sample preparation requirements [46,47,48,49,52].

Emerging POC platforms—such as microfluidic LAMP [28,29,30,31], CRISPR-Cas-based diagnostics [32,33], and MEMS VOC sensors [78,79,80,81,82,83,84]—may be eligible for Food and Drug Administration (FDA) clearance or Emergency Use Authorization (EUA) if supported by multicenter clinical validation and usability studies. However, no such platforms have yet progressed beyond the prototype stage. Reimbursement pathways remain uncertain, particularly for biosensors not yet associated with established Current Procedural Terminology (CPT) codes. While reader-assisted CrAg lateral flow assays have shown clinical promise and could be eligible for POC implementation [53,54], microfluidic BDG or GM platforms, as well as host response panels based on transcriptomics or cytokines [54,55,56,57,58,59,60,61,62,63,64,65,66], would likely require new cost-effectiveness evaluations and health technology assessments. Economic feasibility will be especially important in immunocompromised populations managed in outpatient settings or low-resource hospitals, where high test throughput, sample-type compatibility (e.g., serum, BAL, breath), and device portability are critical for adoption.

While microfluidic and MEMS-based biosensing platforms offer compelling technical advantages—including rapid turnaround time, high analytical sensitivity, and compatibility with point-of-care deployment—their clinical translation and commercialization remain contingent upon broader considerations that extend beyond analytical performance. In particular, cost-effectiveness in real-world healthcare settings, especially in resource-limited environments, represents a critical but underexplored determinant of adoption. Although miniaturization and reduced reagent consumption are often cited as inherent economic benefits of microfluidic systems, the overall cost structure of these platforms is influenced by device fabrication (e.g., cleanroom-based MEMS manufacturing), integration of complex components such as microvalves and sensors, requirement for external readers or signal-processing units, and the need for quality-controlled disposable cartridges. In low- and middle-income settings, where fungal infections impose a significant burden among immunocompromised populations, the affordability of such systems must be evaluated in comparison with existing diagnostic standards, including culture, microscopy, and established antigen assays, which, despite their limitations, remain relatively inexpensive and widely accessible [46,47,48,49,52].

Moreover, the successful implementation of biosensor platforms in routine clinical practice depends on the development of standardized interfaces that ensure interoperability with existing laboratory and clinical workflows. At present, many proposed systems rely on proprietary cartridge formats, custom fluidic architectures, and device-specific readout mechanisms, limiting cross-platform compatibility and scalability. The absence of harmonized standards for sample input, data output, calibration procedures, and connectivity with electronic health record systems further complicates integration into hospital infrastructures, particularly in decentralized or outpatient settings [28,29,30,31,32,33,78,79,80,81,82,83,84]. This fragmentation also poses challenges for regulatory approval and large-scale manufacturing, as each platform must be evaluated independently rather than within a unified technological framework.

In addition, maintenance requirements, device robustness, and user training must be carefully considered, especially in settings where access to technical support and laboratory expertise is limited. Point-of-care systems intended for widespread deployment must therefore demonstrate not only analytical reliability but also operational simplicity, durability under variable environmental conditions, and minimal dependence on specialized personnel. These factors are central to achieving regulatory approvals such as CLIA-waived status and to ensuring sustainable implementation in both high- and low-resource healthcare systems. Consequently, future research should incorporate comprehensive cost-effectiveness analyses, standardized hardware and software interfaces, and usability studies alongside clinical validation, in order to bridge the gap between technological innovation and practical healthcare impact.

## 8. Current Limitations and Future Opportunities

Despite rapid progress in microfluidic and MEMS-based biosensors for fungal diagnostics, several hurdles must be addressed before routine clinical use. Chief among these are the scarcity of multicenter clinical validations in at-risk populations, with most reports still benchtop or single-center proofs-of-concept; even promising noninvasive breath-VOC approaches emphasize the need for more in vivo, clinically powered studies [103,109]. Standardization is another critical gap—particularly for fungal VOCs, where marker panels vary with strain, growth phase, media, host metabolism, and environmental background. Signature sesquiterpenes (e.g., α-/β-trans-bergamotene, trans-geranylacetone) can differentiate invasive aspergillosis in small cohorts, but broader reproducibility across centers and standardized sampling are still required [109,113].

From a sample-handling standpoint, performance in real matrices (serum, BAL, exhaled breath condensate) is limited by biofouling and matrix effects on transducer surfaces, as well as material interactions in microfluidic devices (e.g., hydrophobic small-molecule absorption into PDMS). Antifouling coatings [zwitterionic, peptide, hydrogel, Self-Assembled Monolayer (SAM)-based] and interface engineering improve robustness but are not yet harmonized across platforms [110,111,112].

Practical mitigation strategies to minimize biofouling and matrix effects include the incorporation of upstream sample-conditioning modules (e.g., on-chip plasma separation, inertial or membrane filtration, and size-exclusion structures) to remove cells and macromolecular debris before contact with the sensing surface. Disposable or regenerable capture layers—such as sacrificial hydrogel pads, nanostructured coatings, or replaceable electrode films—can confine non-specific adsorption away from the core transducer and allow periodic renewal. The use of intrinsically low-fouling materials (e.g., fluoropolymers, glass, or zwitterionic coatings) in critical regions of the microchannel, combined with controlled shear rates and optimized flow profiles, further reduces protein adsorption and cell adhesion. For electrochemical and impedance-based sensors, dynamic regeneration protocols (potential pulsing, enzymatic or mild chemical cleaning) and the inclusion of reference channels shielded from analyte contact help distinguish true binding events from non-specific matrix contributions, which is particularly important for label-free mechanical and EIS platforms [114].

Looking forward, three trends are especially promising. First, artificial intelligence/machine-learning-assisted interpretation can learn complex multianalyte patterns from VOC fingerprints, impedance spectra, cytokine panels, or mechanical sensor arrays and has already transformed analogous biosensing and agri-food sensing fields. Jin et al. summarized how classical machine-learning methods (support vector machines, random forests, k-nearest neighbours, artificial neural networks) and deep learning models can be coupled with electrochemical, optical and field effect biosensors to improve sensitivity, selectivity and multiplexed analysis in AI-powered biosensing systems [115]. Astuti et al. demonstrated a metal-oxide gas-sensor array for detecting *E. coli* contamination in chicken meat, where random forest and support vector machine classifiers converted high-dimensional sensor responses into robust sample-level decisions with classification accuracies above 95–98%, illustrating how supervised learning can stabilize and enhance sensor-array readouts under realistic conditions [116]. Jain et al. further showed that Internet-of-medical-things–integrated biosensors for infectious-disease point-of-care testing can be linked to cloud-based analytics and artificial intelligence pipelines, enabling continuous data aggregation, rapid interpretation and personalized infection management [117]. Ferentinos trained deep convolutional neural networks on 87,848 leaf images and achieved overall accuracies above 99% in multi-class plant disease detection, proving that deep learning can reliably extract subtle visual patterns from noisy biological data [118]. Markom et al. implemented an intelligent electronic nose that combined a commercial gas-sensor array with artificial neural networks to distinguish oil palm trees with basal stem rot from healthy trees directly in the field, again demonstrating robust classification from complex volatile profiles [119]. Collectively, these studies show that supervised models such as support vector machines, random forests, gradient-boosted ensembles, artificial neural networks and convolutional neural networks can extract subtle spatiotemporal signatures from high-dimensional biosensor outputs and generalize well to real-world variability. The same families of models, when combined with rigorous cross-validation and external test sets, are directly applicable to fungal VOC profiles, multiplex cytokine signatures and label-free MEMS responses, where they can support both diagnostic classification and performance prediction over time [120]. Second, multiplexed panels that integrate pathogen targets (antigens, nucleic acids, VOCs) with host response markers may help discriminate colonization from invasive disease and support risk-adapted therapy [103]. Third, miniaturized, cartridge-based fungal diagnostics are feasible: for candidemia, T2MR panels already deliver species-level results directly from whole blood in approximately 3–4 h with high accuracy in pooled analyses, illustrating a translational pathway for future MEMS/microfluidic fungal platforms [103,120,121].

Future progress in microfluidic and MEMS-based biosensing for invasive fungal respiratory infections will depend not only on advances in sensor design but also on the integration of intelligent data processing and connected healthcare infrastructures capable of supporting real-time clinical decision-making. In this context, artificial intelligence (AI)-driven signal processing and the Internet of Medical Things (IoMT) represent key enabling technologies that may bridge the persistent gap between benchtop prototypes and bedside application. Biosensor outputs—particularly from multiplexed platforms such as VOC sensor arrays, impedance-based systems, and multianalyte cytokine panels—are inherently high-dimensional and often influenced by biological variability and environmental noise. Machine learning approaches, including supervised classification algorithms and deep learning architectures, have demonstrated the capacity to extract clinically meaningful patterns from such complex datasets, improving both sensitivity and specificity while enabling discrimination between fungal infection, colonization, and non-fungal respiratory conditions [114,115,116,117,118,119,120]. Importantly, these models can be trained on large, heterogeneous datasets incorporating polymicrobial infections, antifungal exposure, and host response variability, thereby enhancing robustness in real-world clinical settings.

Concurrently, the integration of biosensing platforms into IoMT ecosystems allows for seamless data transmission, aggregation, and remote interpretation across healthcare networks. Portable or handheld devices equipped with wireless connectivity can interface with cloud-based analytical pipelines, enabling continuous monitoring, longitudinal data analysis, and rapid clinical feedback without the need for centralized laboratory infrastructure [116]. Such architectures are particularly advantageous in the management of immunocompromised patients, where early detection and dynamic risk stratification are critical, and where access to specialized diagnostic facilities may be limited. Moreover, IoMT-enabled systems facilitate interoperability with electronic health records, supporting personalized treatment decisions and population-level surveillance.

The convergence of biosensing technologies with AI and IoMT therefore offers a realistic pathway toward scalable, point-of-care fungal diagnostics that are not only analytically robust but also clinically actionable. However, this transition will require standardized data formats, validated algorithms across diverse patient populations, and careful consideration of data security, regulatory approval, and cost-effectiveness. Future research should thus prioritize integrated, end-to-end systems that combine sample-to-answer diagnostics with intelligent interpretation and networked healthcare delivery, in order to fully realize the clinical potential of these emerging platforms.

A key opportunity to strengthen the translational trajectory of microfluidic and MEMS-based fungal diagnostics lies in the tighter integration of artificial intelligence (AI) and Internet of Medical Things (IoMT) frameworks with the technical limitations identified earlier, rather than presenting them as parallel or independent innovations. In particular, AI-driven signal processing offers a powerful mechanism to mitigate well-recognized challenges such as matrix effects, biofouling, and limited specificity in complex clinical samples. Biological matrices, including sputum, bronchoalveolar lavage fluid, and serum, introduce significant variability due to the presence of host proteins, cellular debris, extracellular DNA, and coexisting microorganisms, all of which can distort sensor responses and reduce signal-to-noise ratios. Instead of relying solely on hardware-level solutions (e.g., antifouling coatings or sample preprocessing), machine learning algorithms can be trained to recognize and correct for these matrix-induced distortions by identifying consistent patterns across noisy, high-dimensional datasets [115,117].

For example, in VOC-based platforms, supervised learning models can distinguish fungal-specific metabolic signatures from overlapping bacterial or environmental signals, even in polymicrobial contexts, by leveraging multidimensional sensor outputs rather than single-analyte thresholds [86,88,119]. Similarly, in electrochemical, impedance-based, and mechanical biosensors, AI can enhance specificity by filtering non-specific binding signals, compensating for baseline drift, and integrating temporal response dynamics to differentiate true analyte interactions from artefacts [114,115].

Importantly, the integration of these AI-enhanced biosensing systems within IoMT architectures further amplifies their clinical utility by enabling continuous data aggregation, remote calibration, and model updating across distributed healthcare settings. Cloud-connected platforms can incorporate large-scale, real-world datasets—including variations in sample type, antifungal exposure, and patient-specific factors—thereby improving model robustness and generalizability over time [117]. This is particularly relevant for immunocompromised populations, where diagnostic uncertainty is amplified by heterogeneous clinical presentations and frequent co-infections. By embedding adaptive learning algorithms into connected diagnostic ecosystems, biosensor platforms can evolve from static detection tools into dynamic decision-support systems capable of context-aware interpretation. Consequently, the convergence of AI and IoMT should be viewed not merely as a future trend, but as a functional solution to the core analytical and clinical limitations of current biosensing technologies, providing a critical bridge between experimental performance and reliable real-world deployment.

## 9. Conclusions

The rapid evolution of biosensor technologies—ranging from microfluidic nucleic acid amplification to MEMS-based breath analyzers—offers unprecedented potential for point-of-care diagnosis of IFRIs in immunocompromised patients. These platforms can achieve high analytical sensitivity and specificity, rapid turnaround, and compatibility with minimally invasive sample types such as serum, BAL fluid, and exhaled breath. Furthermore, innovations in host response profiling and AI-based signal interpretation may enable more nuanced diagnostic strategies that go beyond pathogen detection alone.

However, despite the technical maturity of many platforms, clinical translation remains limited. As described above, most devices have only been evaluated in small, preclinical, or single-center pilot studies, often without stratification by host immune status or antifungal exposure. Comparative performance against established gold standards such as BAL galactomannan, PCR, or EORTC/MSG-defined criteria is frequently lacking, and real-world validation in high-risk settings—such as HSCT, SOT, and ICU cohorts—remains sparse. Without such evidence, the diagnostic utility of these biosensors cannot yet be generalized to the broader clinical population.

To realize the promise of these technologies, future efforts must prioritize multicenter validation, robust comparator trials, and economic feasibility studies tailored to the unique clinical contexts in which IFRIs occur. Until then, biosensor platforms should be regarded as promising adjuncts rather than replacements for existing diagnostic tools.

## Figures and Tables

**Table 1:** Comparative Summary of Biosensor Platforms for Invasive Fungal Infection Diagnosis.

Biosensor Type	Target Analyte(s)	Detection Method	Sample Type	Turnaround Time	Sensitivity/Specificity	Limit of Detection (LOD) *	Clinical Validation Stage	References
Microfluidic LAMP	Fungal DNA—multicopy rDNA/ITS loci of Aspergillus spp. and mtLSU rRNA of *P. jirovecii*	Isothermal nucleic acid amplification	BAL, sputum	≈30–60 min	Up to 90–95%/90% (small cohorts)	≈10–100 genome copies/reaction (≈1–10 CFU/mL in BAL/sputum)	Preclinical/small clinical studies	[28,29,30,31]
CRISPR–Cas-based microfluidics	Fungal DNA—species-specific A. fumigatus gene/rDNA targets	Cas12/Cas13 cleavage + fluorescence	BAL, sputum	<1 h	≈95%/≈95% (prototype)	≈3–10^2^ genome copies/reaction (platform-dependent)	Proof-of-concept	[32]
Microfluidic ELISA (GM, BDG)	Galactomannan (Aspergillus cell wall antigen); (1→3)-β-D-glucan (panfungal cell-wall polysaccharide)	Immunoassay (fluorescence, colorimetric)	Serum, BAL	≈30 min	Comparable to standard ELISA	GM: low ng/mL to sub-ng/mL; BDG: low-ng/mL range	Early clinical testing	[46,47,48,49,52]
Paper-based BDG assay	(1→3)-β-D-glucan (serum panfungal biomarker)	Colorimetric paper microfluidics + smartphone readout	Serum	<30 min	Moderate; platform-dependent	≈10–100 pg/mL (platform-dependent)	Preclinical	[52]
Digital CrAg LFA	Cryptococcal capsular polysaccharide antigen (CrAg)	Lateral flow + mobile AI-assisted analysis	Serum, CSF	<20 min	>95%/>95%	≈1 ng/mL in serum/CSF	Clinically validated	[53,54]
Host transcriptomics (RT-qPCR)	Host mRNA transcripts (conserved IA gene-expression signatures)	Microfluidic RT-qPCR	Whole blood	≈30–60 min	85–95%/85–90%	≈10–100 transcript copies per target (few pg total RNA per reaction)	Small clinical cohorts	[54,55,56,57,58,59]
Cytokine biosensors (IL-6, IL-8, PTX3)	Host cytokines and pattern recognition protein (IL-6, IL-8, pentraxin-3)	Bead/droplet/electrochemical immunoassay	Serum	<30 min	Variable; typically additive to fungal PCR	Low pg/mL range	Emerging clinical use	[60,61,62,63,64,65,66,69,70,71]
MEMS VOC sensors	Fungal VOCs—especially Aspergillus-derived sesquiterpenes (e.g., α-/β-trans-bergamotene, trans-geranylacetone)	MEMS gas sensors + ML classification	Exhaled breath	≈15–45 min	≈80–95%/≈85–95%	≈ppb to low-ppm concentration range in exhaled breath	Pilot clinical trials	[78,79,80,81,82,83,84]
Mechanical biosensors (cantilevers)	Fungal antigens (e.g., galactomannan, cryptococcal polysaccharide), fungal DNA, and intact spores	Resonance frequency or surface stress shift	BAL, serum	≈10–30 min	High analytical sensitivity; limited clinical data	≈fg–pg mass loading (corresponding to very low ng/mL-equivalent concentrations)	Preclinical	[92,93,94]
Impedance-based biofilm sensors	Surface-attached fungal biofilms (Candida spp., Aspergillus spp.) on device materials	Electrochemical impedance spectroscopy (EIS)	Catheter, urine	≈5–20 min	Good specificity in vitro	≈10^3^–10^4^ CFU/mL equivalent biofilm burden (platform-dependent)	Lab validation only	[99,100,101,102]

* LOD values are reported using units appropriate to each platform (copies/reaction or CFU/mL for nucleic-acid tests, ng/mL or pg/mL for antigen and cytokine assays, ppb/ppm for VOC sensors, and fg–pg mass loading for mechanical biosensors) to facilitate horizontal comparability between technologies. BAL: Bronchoalveolar lavage; CSF: Cerebrospinal fluid; LOD: Limit of detection; IA: Invasive aspergillosis; GM: Galactomannan; BDG: (1 → 3)-β-D-glucan; CrAg: Cryptococcal antigen; fg: Femtogram; pg: Picogram; ng: Nanogram; ppb/ppm: parts per billion/million.

## Data Availability

No new data were created or analyzed in this study.
